# Thermophysical Fingerprinting of Probiotic-Based Products

**DOI:** 10.1038/s41598-019-46469-1

**Published:** 2019-07-10

**Authors:** Hary Razafindralambo, Aurélie Razafindralambo, Christophe Blecker

**Affiliations:** Laboratory of Food Science and Formulation, Department of Gembloux Agro-Bio Tech, University of Liege, Avenue de la Faculté 2B, BAT 140 TERRA Teaching and Research Centre, B-5030 Gembloux, Belgium

**Keywords:** Biopolymers in vivo, Biophysical chemistry

## Abstract

Variability in efficacy and safety is a worldwide concern with commercial probiotics for their growing and inevitable use in food and health sectors. Here, we introduce a probiotic thermophysical fingerprinting methodology using a coupling thermogravimetry and differential scanning calorimetry. Qualitative and quantitative information on the material decomposition and transition phases is provided under heating conditions. By monitoring the changes in both mass and internal energy over temperature and time, a couple of thermal data at the maximum decomposition steps allow the creation of a unique and global product identity, depending on both strain and excipient components. We demonstrate that each powder formulation of monostrain and multistrain from different lots and origins have a unique thermophysical profile. Our approach also provides information on the formulation thermostability and additive/excipient composition. An original fingerprint form is proposed by converting the generated thermal data sequence into a star-like pattern for a perspective library construction.

## Introduction

Nowadays probiotics are worldwide recognized as beneficial live microorganisms for human, animal, and plant species^1–8^. Their use as natural and safe functional ingredients is continuing to grow and cover a wider range of applications for food and non-food products, including fermented foods, dietary supplements, drugs, and cosmetics^9–11^. The success of a probiotic product depends on its quality, safety, and performance. These three criteria are dependent on many factors. Strain specificities and interactions appear in first place since each probiotic strain is unique in its functionalities and performances^12^. Both absolute and relative doses as well as other selective ingredients, such as cryo-protectants and prebiotics incorporated in the formulations, are also among the most important parameters that control their efficacy^13–15^. Beyond strains, doses, and functional enhancer ingredients, the variability in efficacy and safety of a multistrain probiotic formulation on its anti-inflammatory and anti-tumoral effects also depends on manufacturing process conditions^16–18^. Consequently, rapid, cost-effective, and reliable tools are needed for ensuring high standard product quality, safety, and performance. Each starting probiotic material must be first identified at least up to the strain level, because of its strain-specific effects. Moreover, it is absolutely important to fingerprint, that is, to identify by biological, physical, and chemical tests without ambiguity the end-product in order to control and ensure the regularity in overall probiotic properties.

The fingerprinting notion consists in differentiating one thing to others, all belonging to the same category, by analyzing and comparing their unique characteristics with an appropriate technique. Two main approaches are known for fingerpriting probiotics and their activities: the phenotypic techniques based on morphological, biochemical, and physiological methods^19^, and the genotypic ones, which use a universal component or section of nucleic acids and other macromolecules (DNA, RNA and proteins), especially for species identification, or strain differentiating up to clonal level^20,21^. In both cases, the analytical techniques belong to either molecular or non-molecular approaches^22^. However, these gold standard methods are only focused on the strain identity and specificity, and completely ignore the presence of other ingredients, which are commonly used for enhancing the probiotic formulation performance and functionalities. This is one of the reasons why a new approach based on the physical chemistry techniques for probiotic characterization has recently been introduced^23^. In particular, the thermal, surface hydrophobicity, and colloidal properties of probiotic models at the solid (powder) and liquid (dispersion) states have been deeply assessed, revealing a unique global profile/fingerprint for each investigated monostrain and multistrain-based formulations^24^.

As functional ingredients, probiotics are commonly added to food, beverage, dietary supplements, drugs, medical foods, infant formula, and cosmetics^10,12,25^. Formulations contain one (monostrain) or multiple strains (multistrain) for which other ingredients and excipients such as cryoprotectants, prebiotics, and dispersing agents are included^26–28^. Most commercialized probiotic-based products on the market are under homogenous powder form. Such formulations are considered as an assembly of various organic and inorganic compounds, including lipids, proteins, carbohydrates, nucleic acids, and minerals, which mutually interact with each other at nano- and micro-scale level. Nevertheless, all components may also behave as a global and unique physical and chemical material. Assuming such behavior, fingerprinting these products could be possible by thermophysical-based techniques.

Calorimetric-based techniques, which include thermogravimetry (TGA), differential scanning calorimetry (DSC), or the combination of both (TGA-DSC), are one of the most convenient classes of techniques for characterizing and analyzing powder-based products^29,30^. Concerning microorganisms and probiotics in particular, only limited research activities have been undertaken and reported in literature for fingerprinting and related investigation purposes. TGA or DSC analyses have provided the profile and related characteristic data on the decomposition/degradation of spores and vegetative cells for various species of *Bacillus*^31^, the thermal denaturation of whole cells and cell components of *E*. *coli*, as well as the identification of the denaturation events, such as the melting of membrane lipids and ribosomal subunit, protein, and DNA compound denaturation^32^. Recently, we have demonstrated for the first time the coupling TGA-DSC performance in providing different and reproducible decomposition and transition specific characteristics of *L*. *bulgaricus* and *S*. *thermophilus* monostrains, and a multistrain of eight species^24^. It has been concluded from this investigation that such qualitative and quantitative data could serve as probiotic fingerprint, whatever the product complexity. This coupling technique measures the changes in mass and heat flow that allow the characterization of decomposition-degradation and transitions phases of different products such as polymers and minerals compounds^33,34^. This versatile tool presents several advantages, particularly for its high sensitivity, reproducibility and resolution for small sample masses, as well as its high throughput and fast screening possibilities without sample preliminary preparation^35^.

Here, we introduce, describe in details, and demonstrate the fundamental and practical interests of the thermophysical fingerprinting methodology based on a coupling thermogravimetry and differential calorimetry measurement. Qualitative and quantitative information on the material decomposition and transition phases are provided under well-defined heating conditions. The methodology was applied on several powder formulations of various representative monostrain classes of probiotics belonging to *Lactobacillus*, *Bifidobacterium*, *Streptococcus*, *Bacillus* and *Saccharomyces* genera, and multistrains of different lots and origins. An original fingerprint form is proposed by converting the generated thermal data sequence into a star-like pattern.

## Results

**Figure 1:**
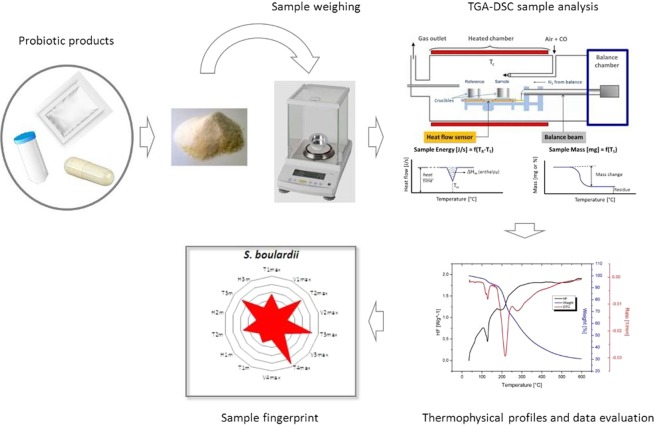
General workflow for fingerprinting probiotic powder formulations by coupling TGA-DSC.

For fingerprinting probiotic powders, we scan thermally the samples from 35 to 600 °C at 5 °C/min under defined conditions as summarized in Table 1, and record simultaneously the changes in their mass and internal energy by using a coupling TGA/DSC technique. The method includes 3 steps without sample pretreatment (Fig. 1): (1) thermal scanning of sample weighed up to 0.1 µg resolution; (2) qualitative profiling and quantitative data processing, and (3) fingerprint generating from both material decomposition and transition characteristics.

**Table 1:** TGA-DSC experimental conditions.

*Experimental conditions*	
Probiotic sample weight	~10 mg
Heating rate	5 °C/min
Temperature range	35–600 °C
Atmosphere	N_2_
Balance resolution	0,1 µg
Crucibles	Aluminum without lid (100 µL)

**Figure 2:**
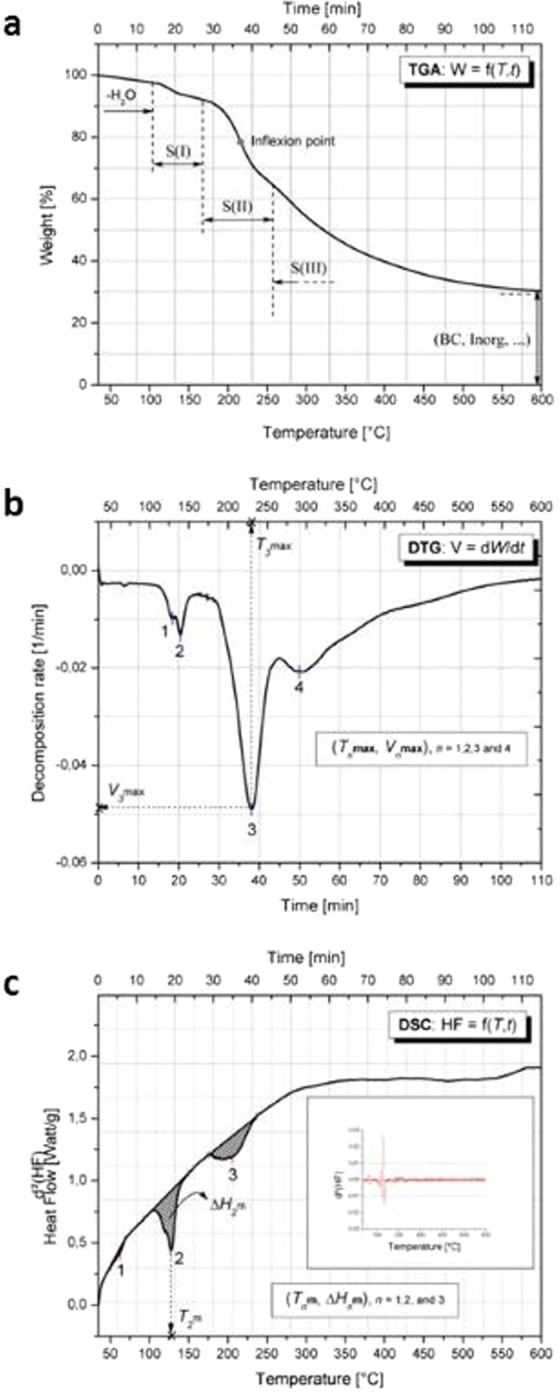
Typical probiotic TGA-DSC generated curves. (**a**) Sample mass *m* variation under temperature scan at 5 °C/min and N_2_ atmosphere. Each decomposition step S(*n*) fits a sigmoid function characterized by an inflexion point. The end of the scan at 600 °C yields the residual mass of carbon black (BC) and inorganic compound material. (**b**) First derivative plot of mass per time unit (d*m*/d*t*) showing the rate of material decomposition. Each peak indicates a decomposition step and the minimum value corresponds to the maximum decomposition rate (*V*_max_) and temperature (*T*_max_), for which the material half-mass is lost. (**c**) Heat flow (HF) variation under temperature scan at 5 °C/min and N_2_ atmosphere. The inset curve is the second derivative of HF vs. temperature used for delimiting and calculating the area (material enthalpy) between the base line and the HF plot.

Each temperature scan takes 2 hours and generates the sample decomposition (TGA curve) and transition (DSC curve) simultaneously, as well as the decomposition rate (DTG curve), through the mass (Fig. 2a) and heat flow (Fig. 2c) variations as a function of temperature and time, and the first derivative of the mass variation (Fig. 2b), respectively. From such profiles, we extract a series of thermophysical data at particular situations of material decomposition and transition for generating a unique fingerprint for each sample.

### Basic and data analysis considerations

#### TGA profile

TGA curves are a succession of sigmoids that represent the evolution of the sample mass (m) as a function of temperature and time, m = f(T, t). Temperature and time are two interdependent variables linked to the heating scan rate. The number of sigmoids indicates the number of material decomposition steps, depending on the temperature scan range.

Each sigmoid is delimited by the initial and final temperatures (Ti, Tf) of mass loss (*L*m), and contains an inflexion point where *L*m reaches 50% of material. All these parameters can be determined by calculating the first derivative dm/dt or dm/dT of m = f(T, t). TGA curves show the profile and complexity of the decomposition process of the investigated sample, and remain especially a qualitative indicator/fingerprint for probiotic-based products.

#### DTG profile

DTG curves are the first derivatives of TGA curves (DTG = dm/dt) that provide the decomposition rate of the material as a function of temperature scan. Such information is consequently dependent on the TGA profiles. DTG curves reach minima at sigmoid inflexion points of TGA curves, where the decomposition rates reach the maximum values. At these points, the maximum decomposition rate (*V*max) and the corresponding temperature (*T*max) can be easily extracted. The couple values (*V*max, *T*max) represent one of the quantitative fingerprints for each probiotic sample. In addition, the DTG curve enables the possible delimitation of each decomposition phase by the determination of the couple (Ti, Tf).

#### DSC profile

DSC curves represent the changes in heat flow (HF) as a function of temperature scan, as represented by the function HF = f(T, t). According to the material reaction compared to a blank reference against the temperature scan, the recorded heat flow varies whether energy is absorbed or released, respectively. A set of peaks with minimum or maximum values are generated during the material process transitions such as melting (solid to liquid), crystallization (liquid to solid), sublimation (solid to vapor), and so on. Each transition peak can be characterized by the corresponding transition temperature (*e*.*g*: the melting temperature *T*m) and the transition enthalpy (*e*.*g*: the melting enthalpy Δ*H*m), representing the internal energy involved during the transition phase. Enthalpy values are calculated by integrating the HF = f(T, t) function for a well-delimited transition zone. The couple (*T*m, Δ*H*m) forms the second quantitative fingerprints of each probiotic sample, which can be associated with the couple (*T*max, *V*max) from TGA curves.

#### Star-like fingerprints

Radar or spider plots of TGA-DSC thermophysical data provide star-like fingerprint patterns of probiotic products. Each pattern represents the series of temperature (°C) – rate (h^−1^) values from TGA decomposition steps, and temperature (°C) – enthalpy (J/g) values from DSC transition phases at the minima of DTG curves. Such a fingerprint is the mirror of the probiotic sample at all situations occurring at significant half-decompositions.

To test the performance of the method, we thermally scanned and fingerprinted two categories of probiotic-based products. The first class contained one strain of probiotic (monostrains), whereas the second one included various mixed strains (multistrains), both categories being commercial products and containing other components such as prebiotics, dispersing agents, and so on (Table 2).

**Table 2:** Monostrain and multistrain probiotic-based product data.

Probiotics	Lots	Excipients/Other ingredients	Origin	Concentration 10^9^ [CFU]
**1. Monostrains**				
*L*. *bulgaricus DM24734*	1102533350	Maltose – SiO_2_	EU	11/g
*S*. *thermophilus DSM24731*	1102603489			91/g
*L*. *rhamnosus GG*	L9301	Inulin-CMM – SiO_2_		100/g
*B*. *longum BB536*	S701176593	CMM – SiO_2_		80/g
*B*. *subtilis CU1*	L1600812	CMM – SiO_2_		50/g
*S*. *boulardii CNCM I-745*	Enterol	Lactose – MgC18		24/g
**2. Multistrains**				
*Multi8 strains* (*MixS8a*)	VMS003NM	Maltose – SiO_2_	EU	102/g
*Multi8 strains* (*MixS8b*)	10151198	Maltose – SiO_2_	US	102/g
*Multi8 strains* (*MixS8c*)	3302E10	Maltose – SiO_2_	US	102/g
*Multi8 strains* (*MixS8d*)	45752	Maltose – SiO_2_	CA	102/g
*Multi6 encapsulated strains* (*MixS6*)	131144	Prebiotic – gelatin – dextrin – triglyceride	US	14/g
*Multi2 strains* (*MixS2*)	10086	Co-Q10 – Vitamins E/B – L-cys – CMM – SiO_2_	EU	12/g

MixS8 strains: *S*. *thermophilus*, *B*. *breve*, *B*. *longum*, *B*. *infantis*, *L*. *acidophilus*, *L*. *plantarum*, *L*. *paracasei*, *L*. *delbrueckii subsp*. *Bulgaricus*; MixS6 strains: *B*. *breve*, *B*. *lactis*, *L*. *acidophilus*, *L*. *plantarum*, *L*. *rhamnosus*, *B*. *subtilis*; MixS2 contains *L*. *fermentum* and red yeast rice (*Monascus purpureus*). CMM: mixture of cellulose microcrystalline, magnesium stearate and maltodextrin. MgC18: magnesium stearate.

### Performance of the method on monostrains

**Figure 3:**
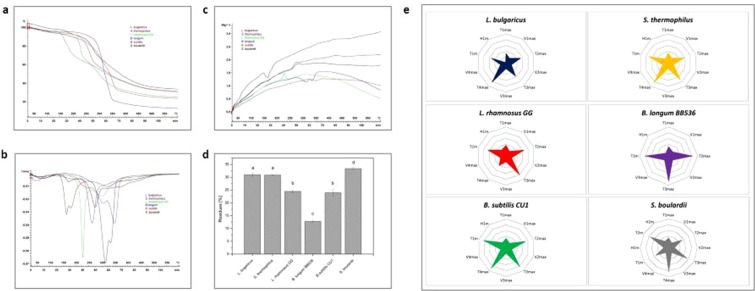
Monostrain fingerprinting results. (**a**) Mass decomposition profiles. (**b**) Mass loss rate profiles; (**c**) heat flow variation. (**c**) Residual mass (mean ± sd, *n* = 3) comparison at 600 °C (different letters indicate significant differences at *p* < 0.05). (**d**) Star-like fingerprints plotted from TGA and DSC thermophysical data at the half-material decompositions for all steps.

For testing the method to monostrains, we thermally analysed a representative of each main class of probiotic, including *Lactobacilli*, *Bididobacteria*, *Bacilli*, *Streptoccocus*, and *Saccharomyces*. Figure 3a,b show the TGA profiles (A), as well as DTG and DSC curves (B) of *L*. *bulgaricus*, *S*. *thermophilus*, *L*. *rhamnosus*, *B*. *longum*, *B*. *subtilus*, and *S*. *boulardii*.

Each sample has its own TGA profile. This is globally represented by one or more sigmoid curves, generated after a linear temperature scan from 34 to 600 °C by 5 °C/min under N_2_ atmosphere. The first parts of TGA curves up to ~100–150 °C are quite similar for all samples. This step represents the elimination of moisture and some volatile components. The second part corresponds to the main decomposition step that occurs between 150 and 350 °C. Differences in the curve features of the monostrain products are observed at this step. The last part of TGA curves from 350 to 600 °C shows the same asymptotically trend, but does not necessarily yield the same amount of material at the end of the temperature scan. Residual compounds at this temperature are mainly constituted by carbon black and inorganic compounds, which are significantly different among monostrain samples (Fig. 3d). These values are comprised between 10 and 35% (w/w) of the initial mass. These materials represent the residual materials from both cells and excipients (SiO_2_, TiO_2_, etc.). In the same way, DTG and DSC curves are also discriminating for each probiotic sample. The main peaks of DTG range between 150 and 350 °C at different positions, and with various intensities. Among representative monostrains scanned, *L*. *bulgaricus* and *B*. *subtilis* show the lowest and highest temperature peak values (*T*max), respectively, whereas *S*. *boulardii* and *B*. *longum* display the lowest and highest peak heights, that is, the maximum rate of mass loss (*V*max), respectively. The DSC curves mainly include endothermic peaks (convexes), but a few ones show exothermic peaks (concaves), particularly for the products containing *S*. *thermophilus*, *L*. *rhamnosus* GG and *S*. *boulardii*. Thermal quantitative data can be generated from TGA-DSC (Supplement Table S1). These thermophysical quantities are determined for establishing the thermal identity or fingerprint of each probiotic-based product, including the material decomposition and transition step number (*n*), rates (*V*max), mass loss (*L*m), temperatures (*T*max and *T*m), enthalpies (Δ*H*m), and residual materials at 600 °C, as detailed in the data analysis procedure section. By considering all couple values (*T*max, *V*max) and (*T*m, Δ*H*m) together, it clearly appears that each monostrain-containing product has its own thermal characteristics, depending on the half-mass loss and the related internal energy changes during the material decomposition and transition steps. Both cell components and excipients in each sample product are expected to contribute to the global thermal profile. Chemically speaking, these compounds are never identical, either qualitatively or quantitatively. Consequently, these thermal data can serve as fingerprints for identifying and controlling any monostrain-containing probiotic product. In order to highlight this statement, such fingerprint data, including all *T*max, *V*max, *T*m and Δ*H*m values from TGA-DSC are plotted in spider or radar form. Figure 3e shows the spider charts of monostrain products. As can be seen, all charts provide a unique star-like pattern for each sample. *B*. *longum*-containing sample has the simplest fingerprint shape, showing only four vertices, whereas that of *S*. *boulardii* is the most complex one, showing six arms and vertices. It simply means that the former probiotic thermal decomposition and transition phases included fewer steps than the latter ones. On the other hand, the fingerprints of *B*. *subtilis* and *S*. *boulardii* are characterized by the longest arms at both T_3_max and T_4_max, respectively, indicating that some steps of decomposition or degradation of such product occurred at the highest temperatures. The other probiotic samples have an intermediate number of vertices.

### Performance of the method on multistrains

**Figure 4:**
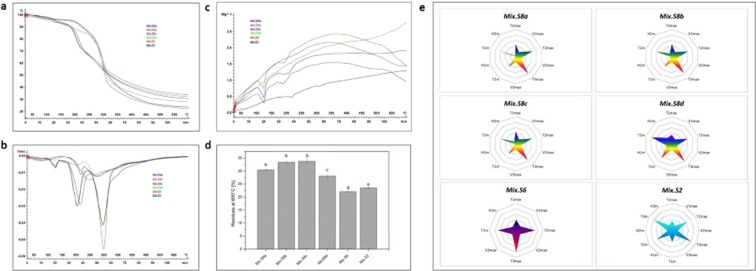
Multistrain fingerprinting results. (**a**) Mass decomposition profiles. (**b**) Mass loss rate profiles; (**c**) and heat flow variation. (**c**) Residual mass (mean ± sd, *n* = 3) comparison at 600 °C (different letters indicate significant differences at *p* < 0.05). (**d**) Star-like fingerprints plotted from TGA and DSC thermophysical data at the half-material decompositions for all steps.

Four multistrain probiotics (MixS8a, MixS8b, MixS8c, MixS8d) were similar formulations from different origins and lots, which were composed by the same mixture of eight strains (*S*. *thermophilus - B*. *breve - B*. *longum - B*. *infantis- L*. *acidophilus - L*. *plantarum - L*. *paracasei - L*. *delbrueckii subsp*. *bulgaricus*). One multistrain-based product contains six bacteria-based probiotics (MixS6), and the other (MixS2) is composed by one bacterium and one yeast strains. Figure 4a–c show their TGA, DTG, and DSC qualitative profiles. Even though the profiles are quite similar for the multistrains MixS8a to MixS8d, which are from different lots but having exactly the same composition, there are some obvious differences in terms of temperatures (*T*max, *T*m), mass loss rate (*V*max), and enthalpy or internal energy (Δ*H*) at certain decomposition and transition steps. MixS6 and MixS2 thermal profiles are clearly different between them, and compared to those of multistrains MixS8. Such differences are attributed to the varieties in strain number and composition, and in excipients as well as in various ingredients which are not necessarily the same, but also in the formulation itself. MixS2 includes, for instance, at least five groups of components such as vitamins (E, B), antioxidants (Co-Q10), and amino acid (L-cystein), besides the excipients, whereas MixS6 contains encapsulated probiotics and prebiotics.

Thermal quantitative data are provided in Table S2 (Supplement Data). Significant differences can also be revealed for the material residues at 600 °C (Fig. 4d), which are less discriminant compared to those of monostrains. The multistrain fingerprints in spider charts or radar graphs are also generated by star-like patterns (Fig. 4d). Their general geometrical shape presents a big similitude, excepted that formulated with encapsulated probiotics. Similar and more regular star fingerprint forms with 5–6 vertices are observed with MixS8 (a, b, c, d) and MixS2, whereas that of MixS6 is simply in cross-like shape.

## Discussion

The coupling TGA-DSC method can be successfully used as a new approach for fingerprinting probiotic-based formulations. It provides both qualitative profile and quantitative data that distinguish monostrain and multistrain-containing powder samples, whatever their composition and complexity, as shown by the representative probiotics characterized in this investigation. The principle is based on the simultaneous monitoring of the sample mass and internal energy under controlled heating conditions, which are unique for each probiotic product. A fingerprint form is suggested in a star-like pattern, which is easily readable and comparable for any probiotic-based product. Compared to other existing approaches based on molecular or non-molecular approaches^22^, the thermophysical fingerprinting method provides a global and unique signature that reflects the thermal behavior of both microorganisms and particular excipients in the powder formulation, but also includes some traits of the manufacturing processes such as the encapsulation. These parameters, non-traceable by the golden standard methods only based on the identification and authentication of strains, play crucial roles in the functionalities and performance of probiotics, either outside or inside the gut. Considering microorganism behaviors, the performance of the method is illustrated by genera such as *B*. *subtilis*, *S*. *thermophilus*, and *S*. *boulardii*. The highest *T*max of *B*. *subtilis* is obviously due to its sporulation, which is the resistant form that occurs during unfavorable conditions. By contrast, the lowest *V*max of *S*. *boulardii* possibly arises from a physical origin related to the bigger volume/mass of yeasts compared to bacteria, but especially for its particular resistance to temperature and acidic stresses for gene overexpression reasons^36^. The exothermic peak in the DSC curve for *S*. *thermophilus* has been previously discussed^24^, and was assigned to the thermophile nature of this strain. Regarding excipients and/or functionality enhancers, the presence of inulin in the sample containing *L*. *rhamnosus* GG, and lactose in *S*. *boulardii*-based product is formally identified by exothermic peaks at high temperatures superior to 200 °C by the formation of more ordered structures such as difructose dianhydrides^37,38^, and the lactose-protein complex formation initiated at 220 °C^39,40^. For similar multistrain samples having quite identical compositions in strains and excipients, differences in fingerprints mainly result from manufacturing processes of the production sites, as reported by different previous investigations with the same type of samples^17,18^. Among its advantages, TGA-DSC coupling technique is fast, highly reproducible, requires only small amount of sample without pretreatment or solvent, and high throughput adapted-operation. Its use in quality standardization and control of probiotic manufacturing is obviously more than useful today, owing to the unprecedented great interest in these beneficial microorganisms for human, animal, and plant life. Beyond the fingerprint establishment, thermal characteristic data can also fundamentally serve as prediction information of probiotic thermostability, and even for predicting their viability and history. This requires furthermore relationship investigations between probiotic thermal properties and viability. Moreover, this coupling method has a potential analytical quantification of each probiotic monostrain ratio in multistrain formulations.

## Conclusion

In summary, we demonstrate and propose in this investigation a new reliable methodology and standard tool for fingerprinting probiotic-based formulations by thermophysical profiling through a wide range of representative samples. The approach is based on the changes in mass and internal energy of mono- or multistrain-containing powders measured by the coupling TGA-DSC under temperature linear scan. Thermophysical data from both decomposition and transition phases of all components in the formulation are used for building an original and unique star-like fingerprint of each probiotic-based product. Compared to the golden standard methods based only on the strain identification and authentication, our methodology has the advantage of providing the identity and traceability of samples through all constituents, including probiotic strains, excipients, and all other ingredients, but also the manufacturing processes used in the formulation. Beyond practical and economic interests, the approach also provides important fundamental information on the chemical composition and product thermostability. A database creation of probiotics available in the market is under investigation for both fundamental, identification and control perspectives.

## Methods

### Probiotics

All probiotics were commercial powders of monostrain or multistrain bacteria and/or yeast purchased or provided by various Companies from Europe and United State. Samples contained various excipients according to the manufacturer formulation and were fingerprinted under this state within unexpired dates. Their composition, origin, lot, and characteristics are listed in Table 1.

### TGA-DSC experiments

TGA-DSC thermal analysis consists in heating a sample while monitoring simultaneously its mass and energy content by gravimetry and heat flow measurements, respectively. The TGA-DSC instrument combines a calorimeter, which is a special furnace for controlling and measuring temperature changes of the material, and a microbalance for mass measurements. Calorimetric analyses were performed with a Mettler-Toledo TGA/DSC1 instrument under defined conditions^24^. A powder sample was weighted with high precision (±0.01 mg), and then deposited onto an aluminum crucible. Runs were performed by monitoring simultaneously the changes in the sample mass (m) and heat flow (HF) as a function of the linear increasing temperature under defined conditions, as summarized in Table 2. Each experiment was carried out at least in triplicate. Raw data were recorded and plotted under TGA and DSC curves for qualitatively profiling the decomposition and transition phase steps. Derivatives (first and second orders) representation and mathematical operations (minima and integration calculations) were performed for generating a couple of thermophysical quantitative data for fingerprinting each probiotic sample. Radar or spider plots were used for generating star-like fingerprint patterns of probiotic products from the series of data at the material half-decomposition (*T*1max, *V*1max, *T*2max, *V*2max, *T*nmax, *V*nmax, *T*1m, Δ*H*1m, *T*2m, Δ*H*2m, *T*nm, Δ*H*nm), where *n* represents the number of decomposition step. STARe software version 16.10 (Schwarzenbach, Switzerland), Microsoft Excel 2010, and Origin pro 8.0 were used for data processing and graphical presentation.

### Statistical analysis

Statistical analysis was performed with the software Origin Pro 8.0 by using the Tukey’s test of the ANOVA 1-way procedure. The means of triplicate thermophysical data between samples were compared at the *p*-value 0.05 signification level.

## Supplementary information

Dataset 1
