# Supplementary material for: Thermophysical Fingerprinting of Probiotic-Based Products
Source: Sci Rep. 2019 Jul 10;9:10011. doi: 10.1038/s41598-019-46469-1 (PMC6620332; doi:10.1038/s41598-019-46469-1)
Supplement: Supplementary file 1 — Dataset 1 [file 41598_2019_46469_MOESM1_ESM.pdf]

Thermophysical Fingerprinting of Probiotic-Based Products

Hary Razafindralambo*, Aurélie Razafindralambo, Christophe Blecker

Laboratory of Food Science and Formulation

Department of Gembloux Agro-Bio Tech, University of Liege

CARE FoodisLife, TERRA Teaching and Research Centre

Avenue de la Faculté d'Agronomie 2B, BAT 140, B-5030 Gembloux, Belgium

* *Corresponding author*

Phone: +32 81 62 21 48

Email: h.razafindralambo@uliege.be

SUPPLEMENT DATA

Table S1: Thermal quantitative data (mean ± sd, n = 3) of probiotic monostrain generated by TGA-DSC technique. Significant differences ($p < 0.05$) are indicated by different superscript letters

	L. bulgaricus	*S. thermophilus*	*L. rhamnosus GG*	*B. longum BB536*	*B. subtilis CU1*	*S. boulardii*
**T1max [°C]**	65,40 ± 0,20 [a]	78,40 ± 1,40 [b,c]	84,20 ± 1,00 [c]	63,60 ± 0,40 [a]	64,20 ± 0,50 [a]	73,60 ± 7,30 [a,c]
**V1max [1/h]**	-0,30 ± 0,05 [a]	-0,15 ± 0,13 [a,b,c]	-0,22 ± 0,00 [a,b,c]	-0,16 ± 0,00 [b,c]	-0,18 ± 0,00 [c]	-0,25 ± 0,01 [a,b,c]
**T2max [°C]**	166,09 ± 0,27 [a]	194,63 ± 1,65 [b]	228,18 ± 0,72 [c]	314,31 ± 0,12 [d]	262,54 ± 0,04 [e]	173,05 ± 0,25 [f]
**V2max [1/h]**	-1,92 ± 0,04 [a]	-0,27 ± 0,00 [b]	-3,87 ± 0,06 [c]	-4,22 ± 0,06 [d]	-0,69 ± 0,00 [e]	-0,41 ± 0,00 [b,f]
**T3max [°C]**	181,69 ± 0,40 [a]	264,58 ± 0,11 [b]	301,89 ± 1,06 [c]	334,13 ± 0,36 [d]	306,53 ± 0,74 [c,e]	275,35 ± 0,35 [f]
**V3max [1/h]**	-1,64 ± 0,02 [a]	-2,32 ± 0,01 [b]	-1,12 ± 0,06 [c]	-3,21 ± 0,04 [d]	-1,48 ± 0,06 [e]	-1,56 ± 0,02 [a,e,f]
**T4max [°C]**	311,61 ± 1,43 [a]	321,87 ± 1,15 [b]	0,00 ± 0,00 [c]	0,00 ± 0,00 [c]	350,72 ± 0,27 [d]	322,36 ± 2,26 [b,e]
**V4max [1/h]**	-0,56 ± 0,01 [a]	-0,63 ± 0,00 [a]	0,00 ± 0,00 [b]	0,00 ± 0,00 [b]	-2,36 ± 0,09 [c]	-0,70 ± 0,01 [a]
**T1m [°C]**	166,63 ± 0,39 [a]	255,05 ± 0,49 [b]	103,37 ± 0,91 [c]	310,33 ± 0,77 [d]	318,91 ± 0,73 [e]	168,64 ± 1,02 [a]
**ΔH1m [J/g]**	-87,36 ± 3,41 [a]	-16,18 ± 0,61 [b]	-1,69 ± 0,15 [c,f]	-31,55 ± 2,09 [d]	11,38 ± 1,00 [e]	-10,19 ± 0,35 [f]
**T2m [°C]**	0,00 ± 0,00 [a]	274,80 ± 0,08 [b]	228,07 ± 1,26 [c]	336,88 ± 0,38 [d]	354,50 ± 0,49 [e]	278,77 ± 1,09 [b,f]
**ΔH2m [J/g]**	0,00 ± 0,00 [a]	24,25 ± 0,69 [b]	26,30 ± 4,16 [b]	-18,63 ± 1,42 [c]	-13,04 ± 2,34 [c]	32,45 ± 0,24 [b,f]
**T3m [°C]**	0,00 ± 0,00 [a]	0,00 ± 0,00 [a]	300,36 ± 0,81 [b]	0,00 ± 0,00 [a]	0,00 ± 0,00 [a]	0,00 ± 0,00 [a]
**ΔH3m [J/g]**	0,00 ± 0,00 [a]	0,00 ± 0,00 [a]	38,03 ± 2,75 [b]	0,00 ± 0,00 [a]	0,00 ± 0,00 [a]	0,00 ± 0,00 [a]

Table S2: Thermal quantitative data (mean ± sd, *n* = 3) of probiotic multistrain generated by TGA-DSC technique. Significant differences ($p < 0.05$) are indicated by different superscript letters

	**Mix.S8a**	**Mix.S8b**	**Mix.S8c**	**Mix.S8d**	**Mix.S6**	**Mix.S2**
**T1max [°C]**	129,10 ± 0,30 [a]	129,20 ± 0,20 [a]	129,30 ± 0,30 [a]	68,50 ± 5,30 [b]	93,10 ± 3,60 [c]	73,90 ± 0,20 [b]
**V1max [1/h]**	-0,47 ± 0,02 [a]	-0,42 ± 0,02 [a]	-0,38 ± 0,00 [a]	-0,23 ± 0,02 [b]	-0,15 ± 0,00 [b]	-0,17 ± 0,00 [b]
**T2max [°C]**	218,17 ± 1,15 [a]	207,32 ± 1,67 [b]	205,11 ± 3,35 [b,c]	204,06 ± 0,90 [b,d]	246,33 ± 0,11 [e]	215,51 ± 0,28 [a]
**V2max [1/h]**	-1,91 ± 0,14 [a]	-1,83 ± 0,05 [a]	-1,90 ± 0,04 [a]	-0,36 ± 0,01 [b]	-0,88 ± 0,01 [c]	-0,64 ± 0,04 [d]
**T3max [°C]**	283,04 ± 4,82 [a]	276,20 ± 0,91 [b]	271,22 ± 2,69 [c]	295, 08 ± 0, 64 [d]	300,10 ± 0,28 [e]	295,55 ± 0,15 [d,e,f]
**V3max [1/h]**	-0,74 ± 0,01 [a]	-0,76 ± 0,05 [a]	-0,68 ± 0,04 [a]	-2,47 ± 0,04 [b]	-3,31 ± 0,03 [c]	-2,49 ± 0,02 [b]
**T4max [°C]**	0,00 ± 0,00 [a]	0,00 ± 0,00 [a]	0,00 ± 0,00 [a]	0,00 ± 0,00 [a]	0,00 ± 0,00 [a]	0,00 ± 0,00 [a]
**V4max [1/h]**	0,00 ± 0,00 [a]	0,00 ± 0,00 [a]	0,00 ± 0,00 [a]	0,00 ± 0,00 [a]	0,00 ± 0,00 [a]	0,00 ± 0,00 [a]
**T1m [°C]**	127,83 ± 0,05 [a]	127,44 ± 0,28 [a]	127,93 ± 0,36 [a]	218,81 ± 0,38 [b]	236,45 ± 5,45 [c]	102,77 ± 0,83 [d]
**∆H1m [J/g]**	-99,88 ± 9,94 [a]	-84,31 ± 3,44 [b]	-85,44 ± 5,13 [b,c]	-2,51 ± 0,32 [d]	-11,97 ± 0,49 [e]	-1,03 ± 0,05 [d]
**T2m [°C]**	208,23 ± 2,08 [a]	197,76 ± 3,10 [b]	195,59 ± 2,53 [b,c]	290,67 ± 3,50 [d]	0,00 ± 0,00 [e]	215,18 ± 0,23 [f]
**∆H2m [J/g]**	-78,75 ± 9,86 [a]	-34,99 ± 3,87 [b]	-48,55 ± 8,19 [b]	36,41 ± 12,78 [c]	0,00 ± 0,00 [d]	-15,40 ± 1,53 [e]
**T3m [°C]**	0,00 ± 0,00 [a]	0,00 ± 0,00 [a]	0,00 ± 0,00 [a]	0,00 ± 0,00 [a]	0,00 ± 0,00 [a]	293,97 ± 5,68 [b]
**∆H3m [J/g]**	0,00 ± 0,00 [a]	0,00 ± 0,00 [a]	0,00 ± 0,00 [a]	0,00 ± 0,00 [a]	0,00 ± 0,00 [a]	11,10 ± 3,74 [b]
